# The respiratory microbiome associated with chronic obstructive pulmonary disease comorbidity in non‐small cell lung cancer

**DOI:** 10.1111/1759-7714.14463

**Published:** 2022-05-17

**Authors:** Masamitsu Shimizu, Akihiko Miyanaga, Masahiro Seike, Kuniko Matsuda, Masaru Matsumoto, Rintaro Noro, Kazue Fujita, Yoko Mano, Nobuhiko Furuya, Kaoru Kubota, Akihiko Gemma

**Affiliations:** ^1^ Department of Pulmonary Medicine and Oncology, Graduate School of Medicine Nippon Medical School Tokyo Japan; ^2^ Department of Clinical Laboratory Medicine, Faculty of Health Science Technology Bunkyo Gakuin University Tokyo Japan

**Keywords:** COPD, ddPCR, lung cancer, microbiome

## Abstract

**Background:**

Research has shown that some microbiomes are linked to cancer. Hence, we hypothesize that alterations in the respiratory microbiome might be associated with lung cancer.

**Methods:**

Through droplet digital polymerase chain reaction analysis, we investigated the abundance of *Acidovorax* in surgically resected primary tumors and corresponding nontumor lung tissues obtained from 50 Japanese patients with non‐small cell lung cancer.

**Results:**

The rate of positivity for *Acidovorax* in tumor and nontumor tissues was 44 and 26%, respectively. The abundance of *Acidovorax* in tumor tissues was significantly higher in patients with nonsquamous cell carcinoma complicated by chronic obstructive pulmonary disease (COPD) and those who relapsed after surgical resection (*p* < 0.05). In tumor tissues, the results of the univariate and multivariate analyses revealed that only COPD exerted a direct effect on the abundance of *Acidovorax* (*p* < 0.05). Furthermore, the presence of *Acidovorax* was high in lung cancer patients with COPD comorbidity (65%) and *TP53* gene mutation; only one of the nontumor tissues was positive for *Acidovorax*. In patients with lung cancer complicated by COPD, *Acidovorax* tended to be present in both the tumor and nontumor areas.

**Conclusions:**

This study identified novel microbiota involved in lung cancer with COPD comorbidity. The results suggested that *Acidovorax* may be a useful biomarker in the screening for lung cancer. Further studies are warranted to validate the clinical significance of the microbiome in a larger independent patient cohort.

## Graphical abstract

This study identified *Acidovorax*, a novel microbiota involved in lung cancer with COPD comorbidity and *TP53* gene mutation using droplet digital polymerase chain reaction. The abundance of *Acidovorax* in tumor tissues was significantly higher in patients with nonsquamous cell carcinoma complicated by COPD and those who relapsed after surgical resection (*p* < 0.05). Alterations in the lung microbiome may be a useful biomarker in the screening for lung cancer.

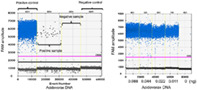

## INTRODUCTION

In individuals with a normal immune status, the lower respiratory tract is generally a sterile environment. However, recent data from 16S rRNA and metagenomic analyses have completely changed the previous perceptions, showing that bacteria, such as *Proteobacteria*, *Firmicutes*, and *Bacteroidetes*, are localized even in the lungs of healthy individuals.^1^, ^2^, ^3^ Moreover, an increasing number of studies reveal an association between pulmonary microbiota (or dysbiosis) and lung diseases. These diseases include chronic obstructive pulmonary disease (COPD), asthma, cystic fibrosis, and idiopathic pulmonary fibrosis (IPF).^4^, ^5^, ^6^, ^7^

Furthermore, some microbiomes have been linked to cancer. For example, infection with *Helicobacter pylori (H. pylori), Fusobacterium nucleatum*, human papilloma virus, and *Escherichia coli* can lead to various types of cancer.^8^ Particularly, *H. pylori* and *Fusobacterium nucleatum* are related to a high risk of gastric cancer and poor prognosis in patients with colorectal cancer, respectively.^9^, ^10^ Nevertheless, further investigation of the relationship between the microbiome and lung cancer is warranted. Although limited, available evidence supports a potential link between lung cancer and alterations in the microbiome found in bronchoalveolar lavage fluid (BALF), lung tissue, sputum, saliva, and fecal samples.^11^, ^12^, ^13^, ^14^ There are notable differences in the microbiomes found in the upper and lower respiratory tracts.^15^, ^16^, ^17^ Also, the analysis of saliva and sputum samples may be influenced by the presence of oral microflora. Therefore, microbiomes should be examined in lung tissues or BALF samples. Previous studies analyzing lung tissue samples have reported that several microbiomes, including *Acidovorax*, are associated with lung cancer.^18^, ^19^ Thus, additional research is required to examine the mechanisms involved in the association of the microbiome with lung cancer. Based on its stability in the long term, the microbiome may be valuable as a diagnostic biomarker and therapeutic target.^20^

The present study focused on the specific microbiome associated with lung cancer. Using droplet digital polymerase chain reaction (ddPCR), we analyzed the abundance of *Acidovorax* in surgically resected primary tumors and nontumor lung tissues obtained from 50 patients with non‐small cell lung cancer (NSCLC). Since low biomass in the bacterial lung tissue microbiome is at the limit of detection, we utilized quantitative PCR (qPCR) 16S bacterial assays in this investigation.

## METHODS

### Patients and tissue samples

We performed a retrospective analysis of 100 patients with NSCLC who underwent surgery at Nippon Medical School Hospital between 2013 and 2018 prior to specimen collection. We also analyzed 50 patients with NSCLC to investigate the presence of an epidermal growth factor receptor (*EGFR*) gene mutation or *ALK* fusion gene. Tissue samples were stored at −80°C in liquid nitrogen. In terms of *EGFR* mutation status, 10 and six patients harbored a deletion in exon 19 and an L858R missense mutation in exon 21, respectively. Histological analysis indicated 35 adenocarcinomas, 11 squamous cell carcinoma (SCC), two large cell carcinoma, and two NSCLC not otherwise specified. A total of 17 patients had COPD comorbidity; 12 and five patients with stages I and II Global Initiative for Chronic Obstructive Lung Disease (GOLD), respectively. The TNM staging system and the American Joint Committee on Cancer Staging Manual (seventh edition) were utilized for TNM staging.

The Institutional Review Board of Nippon Medical School Hospital approved the protocol of this study. All patients provided informed consent, and the study was conducted in accordance with the tenets of the Declaration of Helsinki, 2008.

### Cell culture

Human normal lung fibroblasts, namely human fetal lung fibroblast (HFL‐1), HLF were obtained from the RIKEN RBC Cell Bank and the American Type Culture Collection (ATCC), respectively. In addition, human normal bronchial epithelium, namely BET‐2A and BEAS‐2B were obtained from the ATCC. Cells were amplified and frozen, and one aliquot was thawed for this study. HFL‐1 and HLF cells were routinely screened for the absence of mycoplasma and maintained in RMPI 1640 medium (Sigma–Aldrich) with 10% heat‐inactivated fetal bovine serum and 1% penicillin/streptomycin at 37°C in an incubator containing 5% CO_2_. BET‐2A and BEAS‐2B cells were maintained in BEGM Bullet Kit (Takara B3170) at 37°C in an incubator containing 5% CO_2_.

### DNA preparation

The QIAamp DNA Mini Kit (Qiagen) was used to extract genomic DNA from cell pellets or tissue samples, as previously described.^21^, ^22^ A NanoDrop 2000 spectrophotometer (Thermo Scientific) was employed to quantify the genomic DNA.

### Bacteria and bacterial culture

*Acidovorax temperans* was obtained from BCCM/LMG (Laboratory of Microbiology, Ghent University) for use in qPCR positive control experiments. The bacteria were cultured and maintained in American Type Culture Collection medium 3 nutrient agar/broth for 3 days.

### Detection and quantification of bacterial abundance using ddPCR

The QX200 Droplet Digital PCR system (Bio‐Rad) was employed to quantify the copies and total DNA (RPP30 reaction) of *Acidovorax*. Prime PCR reagents were used in the design of primers and probes (Bio‐Rad). The Unique Assay ID used for *Acidovorax* was dCNS492472816. The MQE Context sequence, which is a peripheral reference sequence containing the amplicon sequence of the assay system, was GGATTAGATACCCTGGTAGTCCACGCCCTAAACGATGTCAACTGGTTGTTGGGTCTTCACTGACTCAGTAACGAAGCTAACGCGTGAAGTTGACCGCCTGGGGAGTACGGCCGCAAGGTTGAA. Similarly, the Unique Assay ID used for RPP30 was dHsaCP2500350. The MQE context sequence was TCGGCCATCAGAAGGAGATGAAGATTGTCTTCCAGCTTCCAAGAAAGCCAAGTGTGAGGGCTGAAAAGAATGCCCCAGTCTCTGTCAGCACTCCCTTCTTCCCTTTTATAGTTCATCAGCCAC. Each reaction (total volume: 20 μl) contained forward and reverse primers/probe sets (1 μl), 1× ddPCR SuperMix for Probes (Bio‐Rad) (10 μl), and bisulfite‐converted DNA. The amount of DNA per well was adjusted to 500 ng. Samples were analyzed using automated droplet generator cartridges (Bio‐Rad) with automated droplet generation oil for probes (Bio‐Rad).

Droplets were added to a 96‐well PCR plate. The reaction was performed using a C1000 touch thermal cycle with 96‐deep well reaction module (Bio‐Rad) as follows: DNA polymerase activation (10 min at 95°C), denaturation (40 cycles of 30 s at 94°C), annealing and extension (1 min at 63°C), DNA polymerase deactivation (10 min at 98°C), and cooling (4°C). The PCR plates were subsequently loaded into a QX200 Droplet Reader (Bio‐Rad), and the presence of *Acidovorax* or RPP30 was determined. The QuantaSoft software (QuantaSoft v1.7.4; Bio‐Rad) was employed for data analysis. All reactions included a blank DNA template control well and bisulfite‐converted control DNA (500 ng). The amount of DNA used for each sample was 10 or 20 ng. For quality control, samples that yielded <100 positive droplets in the RPP30 reaction were excluded. In this study, the number of copies of *Acidovorax* or RPP30 was used for statistical analysis.

### *TP53* gene sequencing and mutation analysis

Genomic DNA extracted from 47 lung cancer tissues was submitted for *TP53*‐targeted sequencing using Sanger sequencing. This was performed following standard procedures, as previously described.^23^ Table S1 lists sequences for forward and reverse primers used in the sequencing assay. Because only the tumor was sequenced, during the scoring of somatic mutations, those deemed to be germline were excluded. These included single‐nucleotide variants present in the single nucleotide polymorphism database with high reported allele frequency (i.e., common polymorphisms). In addition, single‐nucleotide variants in untranslated regions and introns were not considered as their somatic status and functional implications are unclear. The presence of putative somatic exonic and splicing variants was verified using database from The Cancer Genome Atlas and Catalogue of Somatic Mutations in Cancer.

### Statistical analysis

A logistic regression analysis was performed to investigate the associations of bacterial abundances with the clinicopathological characteristics of patients. The correlation between the abundances of bacterial genera was evaluated using Spearman rank correlation analysis. Logistic regression was utilized for the construction of prediction models. To evaluate the diagnostic significance of potential biomarkers, we performed receiver operating characteristic (ROC) curve analysis and calculated the area under the ROC curve (AUC). Statistically significant differences (i.e., *p*‐values < 0.05) versus control were determined using the standard Student's *t*‐test. The SPSS (version 25.0; IBM Corporation) software was used for the analyses.

## RESULTS

### Analysis of *Acidovorax* using the ddPCR assay

**FIGURE 1:**
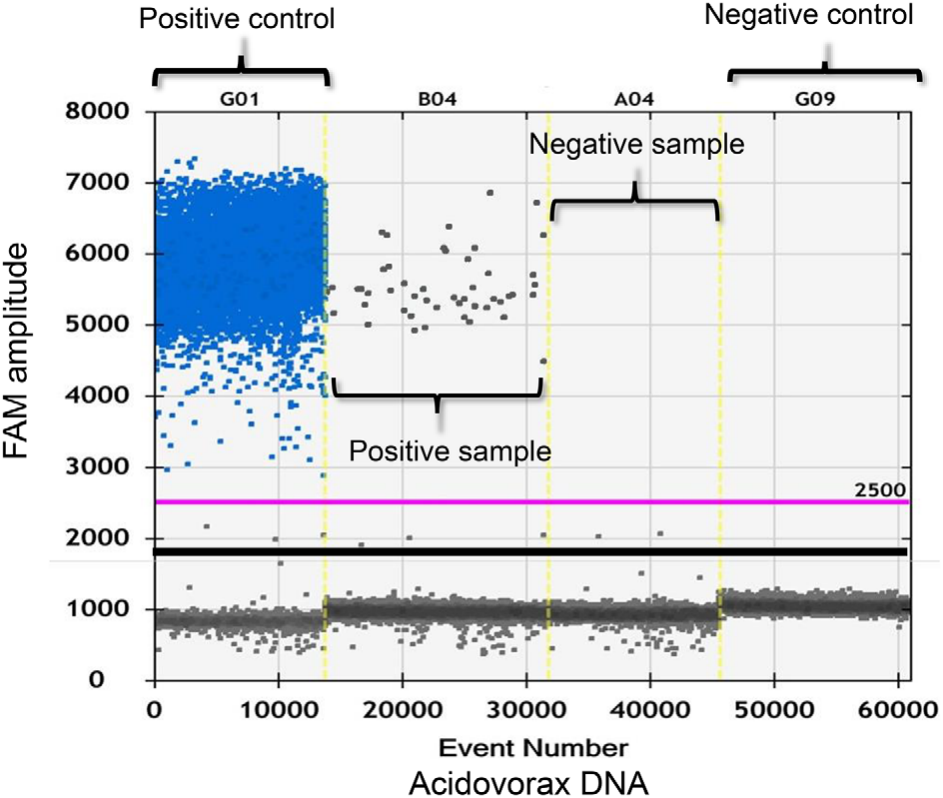
Plot of the ddPCR analysis showing the presence of *Acidovorax* (blue and gray dots indicate positive and negative amplification droplets, respectively). The reaction was specific to the abundance of the *Acidovorax* genera. ddPCR, droplet digital polymerase chain reaction.

The initial experiments aimed to accurately identify the abundance of *Acidovorax* using ddPCR. The sensitivity and analytical range of this method were determined by identifying the lower limit of detection. For this assay, 10‐fold serial dilutions of control DNA (RPP30) in water were prepared. An input DNA amount ≥0.02 ng was sufficient to perform the assay, which showed linearity over the titration series. Through this assay, *Acidovorax* was detectable at a minimum level of 0.025% (Figure S1). Figure 1 shows the DNA abundances of bacteria genera. All the bacterial genera tested using ddPCR generated ≥10 000 droplets per well; therefore, their presence could be quantified in the tissue specimens.

### Abundance of *Acidovorax* in NSCLC

We first investigated the abundance of both *Acidovorax* and total DNA (RPP30) in 50 primary lung tumors and nontumor tissues using ddPCR analysis. Representative results are shown in Figure 1, and the positivity rates for all clinicopathological subtypes are provided in Table 1 (median age: 68 years; males: 33 [66%]; females: 17 [34%]; smokers: 42 [84%]). The histological subtypes were adenocarcinoma (*n* = 35, 70%), SCC (*n* = 11, 22%), and others (*n* = 4, 8%). All patients, except for four patients with pathological stage IV disease, underwent complete tumor resection. Seventeen patients (34%) had lung cancer with COPD comorbidity. Treatment with antibiotics prior to surgery was not administered to any of the patients.

**TABLE 1:** Association of the *Acidovorax* abundances with clinicopathological characteristics of NSCLC patients in tumor tissues

Characteristic	Total	*Acidovorax* of tumor tissue		*p*‐value*
		Positive (%)	Negative (%)	
Gender				
Male	33	14 (42%)	19 (58%)	0.760
Female	17	8 (47%)	9 (53%)	
Age				
<65 years	19	6 (32%)	13 (68%)	0.173
≥65 years	31	16 (55%)	15 (45%)	
Smoking status				
Never smoked	16	6 (37%)	10 (63%)	0.535
Current or former smoker	34	16 (47%)	18 (53%)	
Histological subtype				
Adenocarcinoma	35	16 (46%)	19 (54%)	**0.041**
Squamous cell carcinoma	11	2 (18%)	9 (82%)	
Others	4	4 (100%)	0 (0%)	
Pathological stage^†^				
I, II	30	13 (43%)	17 (57%)	0.910
III, IV	20	9 (45%)	11 (55%)	
COPD				
Absent	33	11 (33%)	22 (67%)	**0.040**
Present	17	11 (65%)	6 (35%)	
*EGFR* gene mutation/ALK fusion gene				
Wild‐type	34	16 (47%)	18 (53%)	0.535
Mutant	16	6 (38%)	10 (62%)	
Recurrence				
Absent	20	5 (25%)	15 (75%)	**0.046**
Present	26	14 (54%)	12 (46%)	
TP53 gene mutation				
Absent	41	14 (34%)	27 (66%)	**0.022**
Present	6	5 (83%)	1 (17%)	

Abbreviations: AC, adenocarcinoma; NSCLC, non‐small cell lung cancer; SCC, squamous cell carcinoma.

* Fisher's exact test. *p*‐values of <0.05 are shown in bold.

^†^ According to the International Union Against Cancer (UICC) TNM Classification of Malignant Tumors, seventh edition (2010).

**FIGURE 2:**
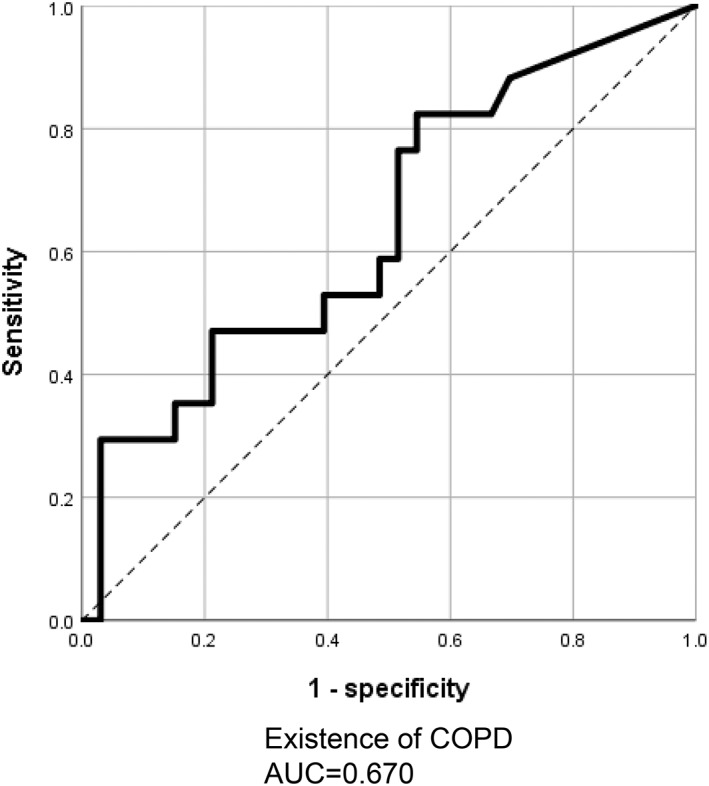
Receiver‐operating characteristic (ROC) curve analysis of the abundance of *Acidovorax* genera in lung tissues obtained from patients with NSCLC. The use of *Acidovorax* as a lung tissue biomarker assisted in detecting lung cancer with COPD comorbidity (AUC: 0.670). AUC, area under the curve; COPD, chronic obstructive pulmonary disease; NSCLC, non‐small cell lung cancer.

Next, the ROC analysis of tumor tissues showed that the diagnostic threshold for the concentration of *Acidovorax* used to screen NSCLC in patients with and without COPD comorbidity was 12.3 copies/well. The sensitivity and specificity of this value were 64.7% and 66.7%, respectively (Figure 2). The AUC was 0.670 (standard error = 0.061; 95% confidence interval = 0.053–0.833; *p* = 0.050). Therefore, we used this threshold as a positive criterion in this analysis. Using the threshold of *Acidovorax* abundance as the cutoff point, its levels were converted into binary variables (i.e., positive and negative).

*Acidovorax* was detected in 46% (*n* = 16/35) and 18% (*n* = 2/11) of patients with adenocarcinoma and SCC, respectively (Table 1). It was also detected in 47% (*n* = 16/34) of current or former smokers, 38% (*n* = 6/16) of patients with an EGFR mutation, and 54% (*n* = 14/26) of those with disease recurrence. The difference in the frequency of *Acidovorax* in SCC and non‐SCC patients was statistically significant (*p* = 0.041, Fisher's exact test). Moreover, the difference in the frequency of relapse after surgical resection was also statistically significant (*p* = 0.046, Fisher's exact test). Furthermore, there was also a statistically significant difference between COPD and non‐COPD patients. (*p* = 0.040, Fisher's exact test). Nevertheless, in nontumor part tissues, there was no significant correlation detected between clinicopathological features and the presence of *Acidovorax* (Table S2). In this study, we investigated *TP53* gene mutations in 47 patients for whom re‐extraction of DNA was possible. The abundance of *Acidovorax* correlated with the *TP53* mutation (Table 1), as 26% (5/19) and 3.6% (1/28) of *Acidovorax*‐positive and *Acidovorax*‐negative cases, respectively, had only the TP53 mutation.

### Comparison of *Acidovorax* abundance in NSCLC tumors and nontumor tissues

**FIGURE 3:**
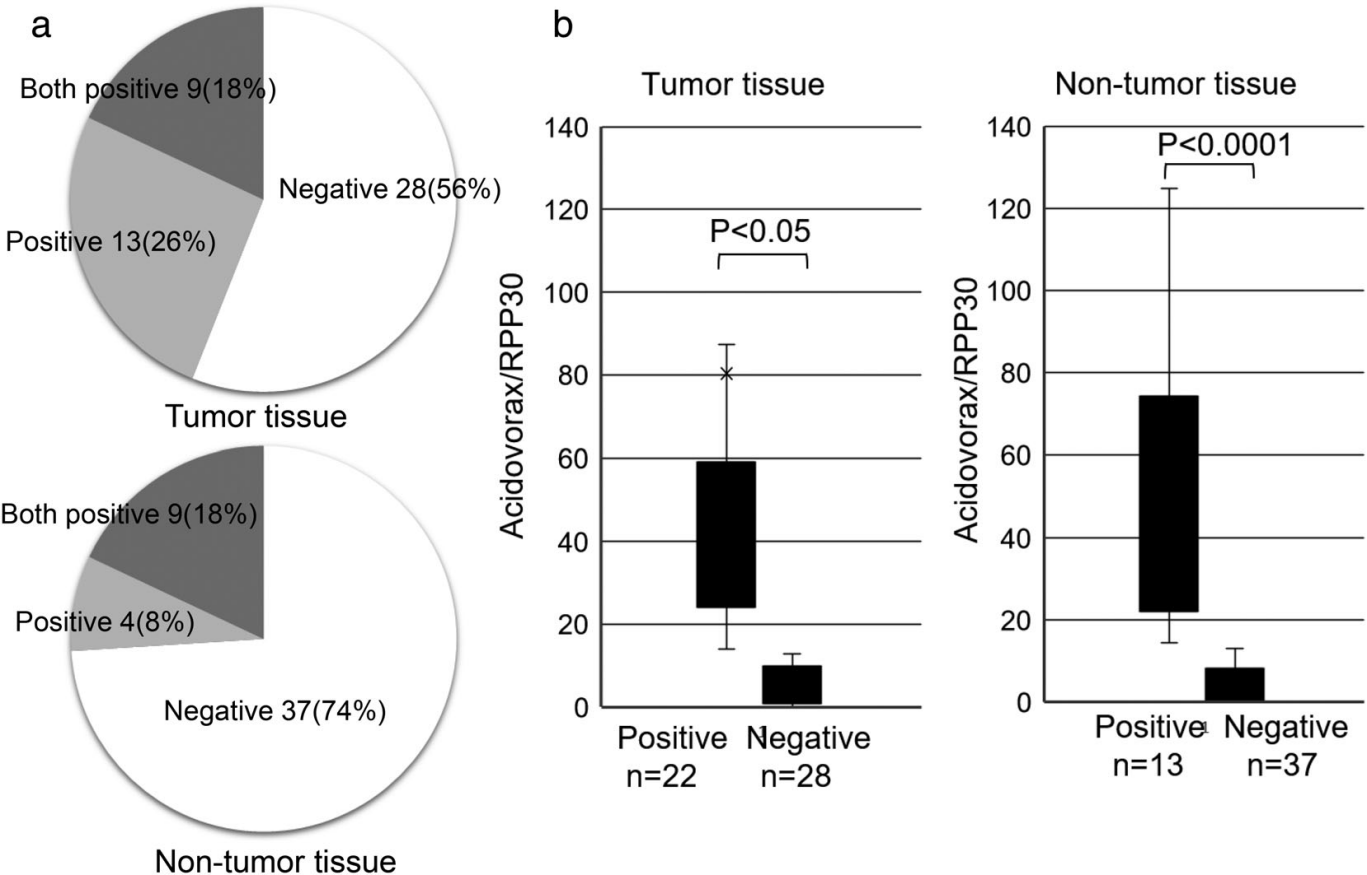
Comparison of the abundance of *Acidovorax* in tumor and nontumor tissues. (a) The rate of positivity for *Acidovorax* in tumor and nontumor tissues. (b) The ratio of *Acidovorax*/RPP30 abundance in tumor and nontumor tissues. The paired *t*‐test was used to evaluate the mean values.

Next, we examined the presence of *Acidovorax* in tumors and nontumor tissues obtained from patients with NSCLC. The positivity rate for *Acidovorax* was 44% (*n* = 22/50) and 26% (*n* = 13/50), respectively; the rate of positivity in both types of samples was 18% (*n* = 9/50) (Figure 3a). We confirmed a statistically significant difference in the number of positive and negative cases of *Acidovorax*/RPP30 between the tumor tissues (*p* < 0.05) and nontumor tissues (*p* < 0.0001) (Figure 3b).

### Abundance of *Acidovorax* is related to NSCLC with COPD comorbidity

Finally, logistic regression analysis was employed to investigate clinical parameters that may affect the abundance of *Acidovorax*. A total of six variables (i.e., sex, age, histology, pathological stage, respiratory comorbidities, and gene mutation) were included as dependent variables (Table 2). The smoking status was excluded as a confounding factor in this analysis. This is because COPD has been closely related to the smoking status, and all registered patients with COPD comorbidity were smokers. In tumor tissues, the results of the univariate analysis showed that COPD (*p* = 0.038) tended to affect the abundance of *Acidovorax*. The multivariate analysis also showed that COPD (*p* = 0.048) and histological subtype (non‐SCC) (*p* = 0.029) had a direct effect on the abundance of *Acidovorax*. In the multivariate analysis of nontumor tissues, COPD (*p* = 0.045) was also associated with the abundance of *Acidovorax* (Table S3). For COPD, positivity for *Acidovorax* was detected in 65% (*n* = 11/17) of tumor tissues, 29% (*n* = 5/17) of both tumor and nontumor tissues, and only one of the nontumor tissues (*n* = 1/17) (Figure S2). Among the lung cancer patients with COPD comorbidity who were positive for *Acidovorax*, seven and four had GOLD stages I and II disease, respectively. Therefore, the abundance of *Acidovorax* in patients with lung cancer may be involved in early‐stage COPD.

**TABLE 2:** Logistic regression in patients with NSCLC in tumor tissues

Variable	*n*	Univariate analysis			Multivariate analysis		
		HR	95% CI	*p*‐value	HR	95% CI	*p*‐value
Gender							
Male/female	33/17	0.829	0.256–2688	0.755	0.404	0.091–1.787	0.232
Age							
<65/≥65 years	19/31	2.311	0.698–7.647	0.170	4.502	0.969–20.914	0.055
Histological subtype							
SCC/non‐SCC	11/39	0.211	0.040–1.106	0.066	0.118	0.018–0.799	**0.029**
Pathological stage							
I, II/III, IV	30/20	1.070	0.342–3.342	0.907	1.838	0.463–7.297	0.387
COPD							
Absent/present	33/17	3.667	1.072–12.55	**0.038**	4.501	1.014–19.989	**0.048**
Gene mutation							
Absent/present	34/16	0.675	0.200–2.277	0.526	0.592	0.129–2.709	0.499

Abbreviations: CI, confidence interval; HR, hazard ratio; SCC, squamous cell carcinoma. *P*‐values of <0.05 are shown in bold.

## DISCUSSION

We determined the abundance of *Acidovorax* in samples of lung tissues derived from patients using qPCR 16S bacterial assays. We confirmed the abundance of *Acidovorax* through ddPCR analysis using its DNA. Subsequently, we investigated the abundance of *Acidovorax* in a variety of NSCLC samples using a newly established ddPCR primer/probe (Figure 1). In tumor and nontumor tissues, the rate of positivity for *Acidovorax* was 44% and 26%, respectively. In the present study, we used DNA extracted from tumor and nontumor areas instead of bronchoalveolar lavage samples from patients with lung cancer. Therefore, it is important to verify the rate of positivity in the future. There was no association between the abundance of *Acidovorax* and the smoking history (Brinkman Index). However, the abundance of *Acidovorax* was linked to an increase in the recurrence of lung cancer after surgery (Table 1). Therefore, we concluded that the abundance of *Acidovorax* is correlated with the risk of lung cancer. In patients with COPD comorbidity, the levels of *Acidovorax* were significantly higher in patients with COPD comorbidity (65%, *p* < 0.05). Furthermore, in this subset of patients, *Acidovorax* was detected even in nontumor tissues (*n* = 5/17, 29%). Our results showed that non‐SCC had a more pronounced effect on the abundance of *Acidovorax* than SCC (*p* = 0.029) (Table 2); however, *Acidovorax* was not significantly increased in SCC. These results indicated that the abundance of *Acidovorax* may not be a biomarker for a specific histological subtype; instead, it appears to be associated with the malignant progression of NSCLC with COPD comorbidity.

It has been reported that the levels of *Acidovorax* are elevated in NSCLC, including cases involving the TP53 mutation pathway.^18^ Furthermore, a significant increase was observed in the volume of lung tumors in mice inoculated with *Acidovorax temperans*. Based on this finding, *Acidovorax* may contribute to lung carcinogenesis in the presence of activated Kras and mutant p53. Therefore, this microbiome may promote the occurrence and progression of disease.^18^ Studies analyzing BALF samples detected *Acidovorax* in both patients with lung cancer and nonmalignant pulmonary diseases.^24^ Notably, alterations in the microbiome may be partly responsible for tumorigenesis or the occurrence of secondary tumors. The present study further suggests that *Acidovorax* may be a biomarker for lung cancer with COPD comorbidity.

Smokers with COPD are at an increased risk (3–10‐fold higher) of developing lung cancer versus those without emphysema.^25^ Numerous epidemiological studies have associated COPD with lung cancer, regardless of the extent of smoking.^26^ SCC is the most frequent histological type among patients with COPD comorbidity. However, when stratified according to the Global Initiative for GOLD stage of COPD, adenocarcinoma was a more frequent histological subtype than SCC in patients with GOLD stage I disease.^27^ In this study, the number of SCC patients with COPD comorbidity who were positive for *Acidovorax* was low due to the greater proportion of patients with GOLD stage I versus stage II disease. Genome‐wide association studies revealed overlapping genetic susceptibility loci for lung cancer, smoking behavior, COPD, and pulmonary function.^28^ However, there is considerable unexplained variation among individuals in terms of susceptibility for progression from COPD to lung cancer. Thus far, there are no studies investigating the coexistence of lung cancer and COPD using lung cancer tissues; nevertheless, sequencing of the 16S rRNA gene has been conducted using nontumor tissues. Previous research reported that the abundance of *Proteobacteria* (i.e., *Acinetobacter* and *Acidovorax*) was significantly lower, whereas that of *Firmicutes* (*Streptococcus*) and *Bacteroidetes* (*Prevotella*) was higher in patients with lung cancer with or without emphysema versus those with emphysema.^19^ Several studies revealed that the gut microbiota affects the response of patients with cancer to immunotherapy.^29^, ^30^ According to Tsay et al., the lower airway microbiota is commonly enriched by oral commensals, and some of these bacteria affect tumor progression and prognosis by triggering host transcriptomic signatures associated with carcinogenesis.^31^

Most bacterial genera were not detectable by conventional PCR, suggesting that the levels of the microbiome were low in the clinical specimens. Therefore, we used ddPCR, which is more sensitive than conventional PCR, to measure the bacteria. Based on the ddPCR data, the abundance of *Acidovorax* has almost zero copy in normal and lung cancer cells (Figure S3). In contrast, it was >12.3 copies/well in lung cancer tissues obtained from patients with lung cancer, which we judged to be *Acidovorax*‐positive. Similarly, Leng et al. reported that the sputum microbiome may provide noninvasive biomarkers for the early detection and classification of NSCLC using ddPCR.^32^ Therefore, the bacteria may not be present in the normal specimens. The presence of this microbiome could be used as a biomarker of lung cancer.

This study was characterized by several limitations. First, the study involved a small sample size. In the future, we plan to prospectively validate the present findings in a large cohort. Second, we only assessed *Acidovorax* genera that had been previously associated with lung cancer. Although the results are meaningful, the exact association of this bacterial flora with lung cancer remains unclear. Third, we found that the non‐SCC histological subtype was associated with the abundance of *Acidovorax*. However, there was no correlation between the abundance of *Acidovorax* and the SCC subtype (i.e., the most common histological type in lung cancer patients with COPD comorbidity). In this study, the positive rate for *Acidovorax* in patients with SCC was low (18%), and most patients had early GOLD stage disease. *Acidovorax* may also be a preferable risk factor for lung cancer with COPD versus others, such as the extent of smoking and the SCC histological subtype. Finally, this study included only Japanese patients; hence, these findings may not be generalizable to other ethnic groups.

Based on the present results, alterations in the lung microbiome may be a useful biomarker in the screening for lung cancer. Further investigation is warranted to validate the present evidence and identify additional bacterial biomarkers.

## CONFLICT OF INTEREST

The authors have no potential conflicts of interest to disclose.

## Supporting information


**Figure S1.** Plot of the ddPCR analysis showing *Acidovorax* in serial dilutions. Blue and gray dots denote positive and negative amplification droplets, respectively. ddPCR, droplet digital polymerase chain reaction
**Figure S2.** Rate of positivity for *Acidovorax* in tumor and nontumor tissues. (A) 50 cases of lung cancer. (B) 17 cases of lung cancer with COPD comorbidity. COPD, chronic obstructive pulmonary disease
**Figure S3.** Plot of the ddPCR analysis showing the presence of *Acidovorax* (blue and gray dots indicate positive and negative amplification droplets, respectively) in human normal lung fibroblasts and bronchial epithelium. ddPCR, droplet digital polymerase chain reaction

**Table S1.** List of primers and targets for TP53 sequences
**Table S2.** Association of the Acidovorax abundances with clinicopathological characteristics of NSCLC patients in nontumor tissues
**Table S3.** Logistic regression in patients with NSCLC in nontumor tissues
