# Supplementary material for: The respiratory microbiome associated with chronic obstructive pulmonary disease comorbidity in non‐small cell lung cancer
Source: Thorac Cancer. 2022 May 17;13(13):1940–7. doi: 10.1111/1759-7714.14463 (PMC9250845; doi:10.1111/1759-7714.14463)
Supplement: Supplementary file 1 — Figure S1. Plot of the ddPCR analysis showing Acidovorax in serial dilutions. Blue and gray dots denote positive and negative amplification droplets, respectively. ddPCR, droplet digital polymerase chain reaction Figure S2. Rate of positivity for Acidovorax in tumor and nontumor tissues. (A) 50 cases of lung cancer. (B) 17 cases of lung cancer with COPD comorbidity. COPD, chronic obstructive pulmonary disease Figure S3. Plot of the ddPCR analysis showing the presence of Acidovorax (blue and gray dots indicate positive and negative amplification droplets, respectively) in human normal lung fibroblasts and bronchial epithelium. ddPCR, droplet digital polymerase chain reaction [file TCA-13-1940-s002.pptx]

## Slide 1
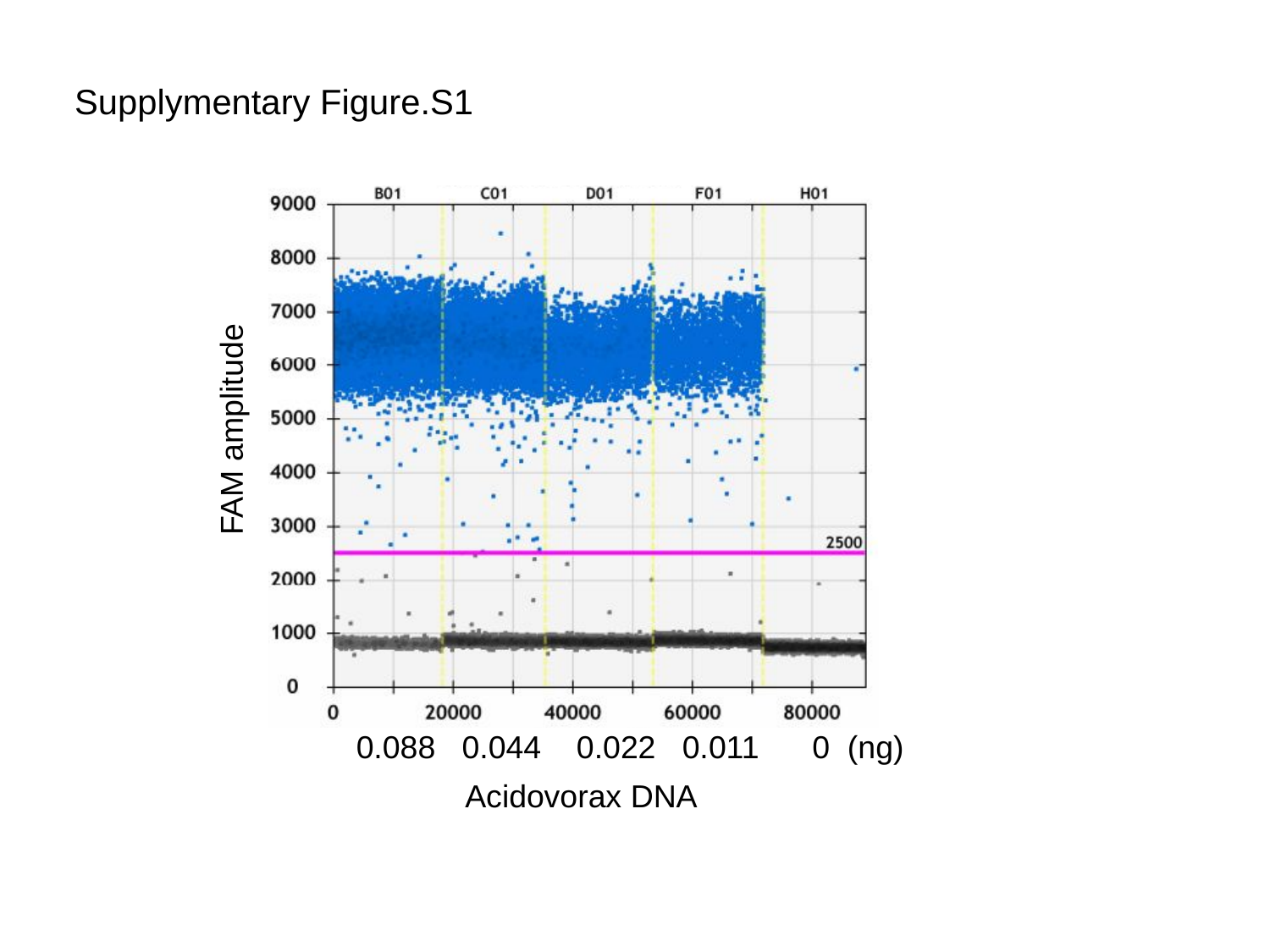

Supplymentary Figure.S1
FAM amplitude
0.088 0.044 0.022 0.011 0 (ng)
Acidovorax DNA

## Slide 2
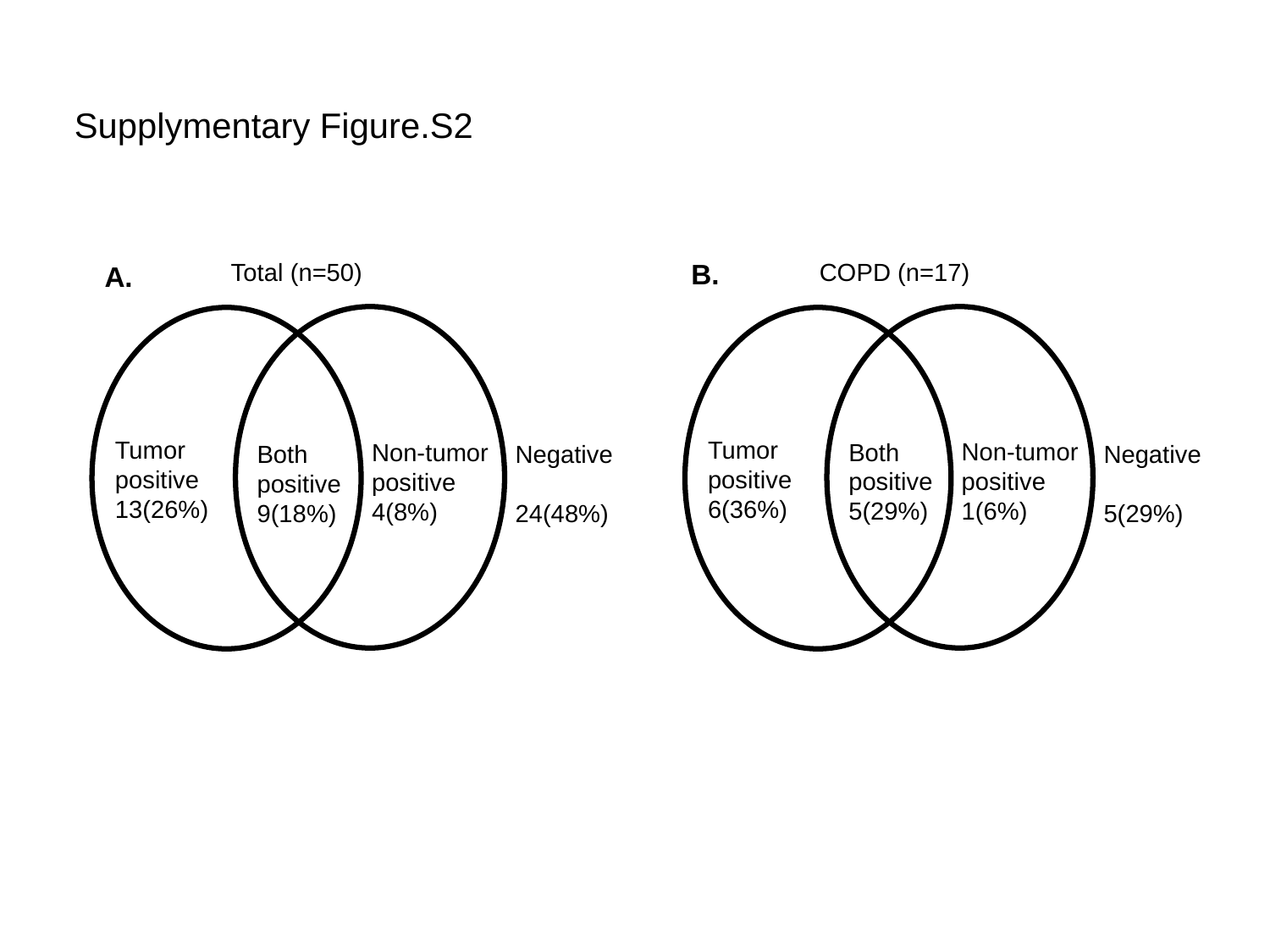

Supplymentary Figure.S2
Total (n=50)
Tumor positive
13(26%)
Non-tumor positive
4(8%)
Both positive
9(18%)
Negative
24(48%)
A.
B.
COPD (n=17)
Tumor positive
6(36%)
Non-tumor positive
1(6%)
Both positive
5(29%)
Negative
5(29%)

## Slide 3
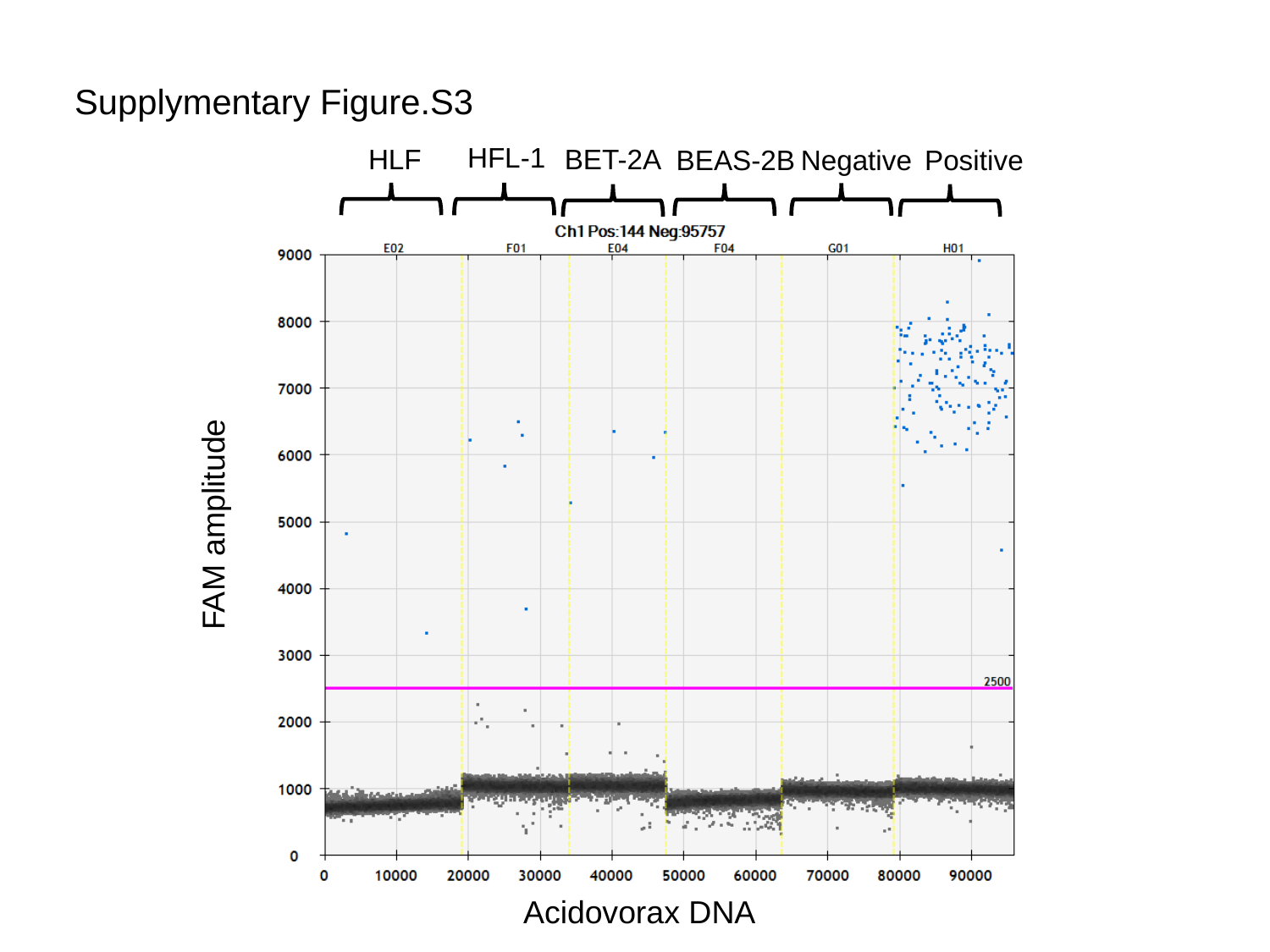

Supplymentary Figure.S3
HFL-1
BET-2A
HLF
BEAS-2B
Negative
Positive
FAM amplitude
Acidovorax DNA
