# Supplementary material for: The respiratory microbiome associated with chronic obstructive pulmonary disease comorbidity in non‐small cell lung cancer
Source: Thorac Cancer. 2022 May 17;13(13):1940–7. doi: 10.1111/1759-7714.14463 (PMC9250845; doi:10.1111/1759-7714.14463)
Supplement: Supplementary file 2 — Table S1. List of primers and targets for TP53 sequences Table S2. Association of the Acidovorax abundances with clinicopathological characteristics of NSCLC patients in nontumor tissues Table S3. Logistic regression in patients with NSCLC in nontumor tissues [file TCA-13-1940-s001.docx]

**Supplementary Table S1. List of primers and targets for TP53 sequences**

| **No** | **Primer sequence**  **(5'-3')-F** | **Primer sequence**  **(5'-3')-R** | **Target Resion** | **Product**  **(bp)** |
| --- | --- | --- | --- | --- |
| 1 | tcccacaggtctctgctagg | tggaagtgtctcatgctgga | chr17:7579765+7579981 | 217 |
| 2 | aaaagagcagtcagaggacc | ccatgggactgactttctgc | chr17:7579604+7579754 | 151 |
| 3 | caggcattgaagtctcatgg | gaagacccaggtccagatga | chr17:7579267+7579521 | 255 |
| 4 | ttctgggaagggacagaaga | cctggtcctctgactgctct | chr17:7579385+7579626 | 242 |
| 5 | gccctgtcgtctctccag | gccctgactttcaactctgtct | chr17:7578339+7578593 | 255 |
| 6 | cttaacccctcctcccagag | ctgctcagatagcgatggtg | chr17:7578137+7578387 | 251 |
| 7 | gggtcagaggcaagcagag | ttgggcctgtgttatctcct | chr17:7577433+7577630 | 198 |
| 8 | gcttcttgtcctgcttgctt | ggtggttgggagtagatgga | chr17:7576997+7577251 | 255 |
| 9 | ccccaattgcaggtaaaaca | ggagaccaagggtgcagtta | chr17:7576759+7576990 | 232 |
| 10 | aggctaagctatgatgttcctt | caatggctcctggttgtagc | chr17:7576470+7576724 | 255 |
| 11 | gaaggcaggatgagaatgga | acttctccccctcctctgtt | chr17:7573842+7574060 | 219 |
| 12 | caagggttcaaagacccaaa | tgtcatctctcctccctgct | chr17:7572838+7573042 | 205 |
| 13 | tccaatggatccactcacag | atccccacttttcctcttgc | chr17:7579821+7579962 | 142 |
| 14 | ggtgaaaagagcagtcagagg | cgaaaattccatgggactga | chr17:7579600+7579762 | 163 |
| 15 | gcattgaagtctcatggaagc | gaagacccaggtccagatga | chr17:7579270+7579521 | 252 |
| 16 | ctgggaagggacagaagatg | gacctggtcctctgactgct | chr17:7579387+7579628 | 242 |
| 17 | aaccagccctgtcgtctct | tgtctccttcctcttcctacag | chr17:7578334+7578576 | 243 |
| 18 | aaccacccttaacccctcct | caggcctctgattcctcact | chr17:7578130+7578321 | 192 |
| 19 | tggaagaaatcggtaagaggtg | cttgggcctgtgttatctcc | chr17:7577404+7577631 | 228 |
| 20 | accgcttcttgtcctgctt | gggagtagatggagcctggt | chr17:7576994+7577244 | 251 |
| 21 | gaaaacggcattttgagtgtt | aagggtgcagttatgcctca | chr17:7576787+7576983 | 197 |
| 22 | ggctaagctatgatgttcctt | acaatggctcctggttgtag | chr17:7576471+7576725 | 255 |
| 23 | ggaatcctatggctttccaa | ttctccccctcctctgttg | chr17:7573859+7574058 | 200 |
| 24 | cagtggggaacaagaagtgg | tcatctctcctccctgcttc | chr17:7572901+7573040 | 140 |

**Supplymentary Table S2. Association of the Acidovorax abundances with clinicopathological characteristics of NSCLC patients in non-tumor tissues**

|  |  | Acidovorax of non-tumor tissue | |  |
| --- | --- | --- | --- | --- |
| **Characteristic** | **Total** | **Positive (%)** | **Negative (%)** | ***P* value*** |
| Gender  Male  Female | 33  17 | 7 (21%)  6 (35%) | 26 (79%)  11 (65%) | 0.292 |
| Age  <65 yr  ≥65 yr | 19  31 | 4 (21%)  9 (29%) | 15 (79%)  22 (71%) | 0.542 |
| Smoking status  Never smoked  Current or former smoker | 16  34 | 4 (25%)  9 (26%) | 12 (75%)  25 (74%) | 0.914 |
| Histological subtype  adenocarcinoma  squamous cell carcinoma  others | 35  11  4 | 7 (20%)  3 (27%)  3 (75%) | 28 (80%)  8 (73%)  1 (25%) | 0.915 |
| Pathological stage†  I, II  III, IV | 30  20 | 6 (20%)  7 (35%) | 24 (80%)  13 (65%) | 0.245 |
| COPD  Absent  Present | 33  17 | 7 (21%)  6 (35%) | 26 (79%)  11 (65%) | 0.292 |
| EGFR gene mutation/ ALK fusion gene  Wild-type  Mutant | 34  16 | 7 (21%)  6 (38%) | 27 (79%)  10 (62%) | 0.211 |
| Recurrence  Absent  Present | 20  26 | 6 (30%)  7 (27%) | 14 (70%)  19 (63%) | 0.823 |

*Fisher’s exact test. *P* values of <0.05 are shown in bold. †According to the International Union Against Cancer (UICC) TNM Classification of Malignant Tumours, 7th edition (2010).

**Abbreviations:** AC, adenocarcinoma; SCC, squamous cell carcinoma.

**Supplymentary Table S3. Logistic regression in patients with NSCLC in non-tumor tissues**

|  |  | Univariate analysis | | |  | Multivariate analysis | | |
| --- | --- | --- | --- | --- | --- | --- | --- | --- |
| **Variable** | ***n*** | **HR** | **95% CI** | ***P* value** |  | **HR** | **95% CI** | ***P* value** |
| **Gender**  Female/male | 17/33 | 0.494 | 0.135-1.808 | 0.287 |  | 0.424 | 0.094-1.923 | 0.266 |
| **Age**  < 65/ ≥65 yr | 19/31 | 1.534 | 0.398-5.908 | 0.534 |  | 2.150 | 0.457-10.12 | 0.333 |
| **Histological subtype**  SCC/non-SCC | 11/39 | 1.087 | 0.240-4.918 | 0.913 |  | 1.681 | 0.250-11.29 | 0.593 |
| **Pathological stage**  I, II/III, IV | 30/20 | 2.154 | 0.597-7.765 | 0.241 |  | 3.426 | 0.764-15.37 | 0.108 |
| **COPD**  Absent/present | 33/17 | 2.026 | 0.553-7.422 | 0.287 |  | 6.111 | 1.041-35.87 | **0.045** |
| **Gene mutation**  Absent/present | 34/16 | 2.314 | 0.625-8.575 | 0.209 |  | 4.538 | 0.775-26.588 | 0.094 |

**Abbreviations:** HR, hazard ratio; CI, confidence interval; SCC, Squamous cell carcinoma.
